# A Potential Involvement of Anandamide in the Modulation of HO/NOS Systems: Women, Menopause, and “Medical Cannabinoids”

**DOI:** 10.3390/ijms21228801

**Published:** 2020-11-20

**Authors:** Renáta Szabó, Denise Börzsei, Zsuzsanna Szabó, Alexandra Hoffmann, István Zupkó, Dániel Priksz, Krisztina Kupai, Csaba Varga, Anikó Pósa

**Affiliations:** 1 Department of Physiology, Anatomy and Neuroscience, Faculty of Science and Informatics, University of Szeged, H-6726 Szeged, Hungary; szaborenata88@gmail.com (R.S.); borzseidenise@gmail.com (D.B.); szzsuzsi15@gmail.com (Z.S.); hoffmannalexandra1228@gmail.com (A.H.); kupai@bio.u-szeged.hu (K.K.); vacs@bio.u-szeged.hu (C.V.); 2 Interdisciplinary Excellence Centre, Department of Physiology, Anatomy and Neuroscience, University of Szeged, H-6726 Szeged, Hungary; 3 Department of Pharmacodynamics and Biopharmacy, University of Szeged, H-6720 Szeged, Hungary; zupko@pharm.u-szeged.hu; 4 Department of Pharmacology and Pharmacotherapy, Faculty of Medicine, University of Debrecen, H-4032 Debrecen, Hungary; priksz.daniel@pharm.unideb.hu

**Keywords:** anandamide, nitric oxide synthase, heme oxygenase, cardiovascular system

## Abstract

Endocannabinoids and their receptors are present in the cardiovascular system; however, their actions under different pathological conditions remain controversial. The aim of our study was to examine the effects of anandamide (AEA) on heme oxygenase (HO) and nitric oxide synthase (NOS) systems in an estrogen-depleted rat model. Sham-operated (SO) and surgically induced estrogen-deficient (OVX) female Wistar rats were used. During a two-week period, a group of OVX rats received 0.1 mg/kg estrogen (E_2_) per os, while AEA-induced alterations were analyzed after two weeks of AEA treatment at the dose of 1.0 mg/kg. At the end of the experiment, cardiac activity and expression of HO and NOS enzymes, content of cannabinoid 1 receptor, as well as concentrations of transient potential vanilloid 1 (TRPV1) and calcitonin gene-related peptide (CGRP) were measured. Our results show that estrogen withdrawal caused a significant decrease in both NOS and HO systems, and a similar tendency was observed regarding the TRPV1/CGRP pathway. Two weeks of either AEA or E_2_ treatment restored the adverse changes; however, the combined administration of these two molecules did not result in a further improvement. In light of the potential relationship between AEA and HO/NOS systems, AEA-induced upregulation of HO/NOS enzymes may be a therapeutic strategy in estrogen-deficient conditions.

## 1. Introduction

Epidemiological studies support the strong correlation between endogenous estrogen level and cardiovascular health. The incidence and prevalence of cardiovascular diseases (CVDs) are much lower in premenopausal women compared to age-matched men, and this sex advantage decreases significantly at the onset of menopause-induced estrogen loss [1,2]. Beside adverse metabolic changes and oxidative damages, estrogen withdrawal also increases sympathetic tone and blood pressure, as well as results in endothelial dysfunction and vascular inflammation, thus exerting a negative impact on vascular homeostasis [3].

A growing body of evidence shows that hormone replacement therapy (HRT) decreases the risk of CVDs and reduces mortality in postmenopausal women with heart disease, which is underpinned by the beneficial effects of HRT on vascular tone regulation [4,5]. Human and experimental studies prove that estrogen replacement therapy enhances coronary flow and decreases both coronary resistance and peripheral vascular tone via enhancing nitric oxide (NO) production [6,7]. With activation of soluble guanylate cyclase (sGC), NO increases the cellular level of cyclic guanosine monophosphate (cGMP) that mediates NO-dependent dilatation. Similar to the nitric oxide synthase (NOS)/NO system, great attention has been placed on the protective role of heme oxygenase (HO) in hemodynamic regulation [8]. HO catalyzes the oxidative degradation of heme to biliverdin, free iron, and carbon monoxide (CO). CO, like NO, leads to the elevation of cGMP levels that results in vasorelaxation. Many studies reported that the cardiovascular effects of the HO/CO system are similar, at least in part, to those of the NOS/NO system, which indicates a possible relationship between their pathways [9]. Beside estrogen-induced activation of NOS/HO systems, there are myriad pathways and mechanisms that suggest the involvement of enhanced NO/CO release in vascular dilatation and cardiac health.

The latest data confirm that endocannabinoids are expressed throughout the cardiovascular system and play a role in the regulation of contractility and vascular tone related to cardiac pathologies [10]. One of the first identified endocannabinoids is *N*-arachidonoyl ethanolamine (anandamide, AEA), which decreases cardiac noradrenaline release and causes endothelium-dependent vasorelaxation via enhancing NO release. Although the activation of the endocannabinoid system (ECS) has paradoxical characteristics, both beneficial and detrimental, it is verified that the ECS is activated as a compensatory mechanism in pathological conditions, in which arterial pressure and cardiac contractility are changed or damaged [11]. AEA-induced vasorelaxation can be mediated via cannabinoid 1 receptors (CB1R) or alternative receptors, such as transient receptor potential vanilloid 1 receptor (TRPV1), which is a ligand-gated ion channel [11,12].

In light of these cardiovascular regulatory mechanisms, we hypothesized that the HO/CO system, similar to NOS/NO, possesses a mediating role in AEA-induced cardiovascular changes. The purpose of our work was to examine the cardiovascular impact of AEA administration on NOS/HO systems as well as on the TRPV1/CGRP pathway in different, estrogen-saturated conditions and to compare these responses to the effects of estrogen hormone therapy.

In our experimental design, female Wistar rats were subjected either to ovariectomy surgery (OVX) to induce an estrogen-deficient condition or to a sham operation (SO) to create a similar stress situation (control group). Both estrogen (E_2_) replacement therapy and AEA treatment regulate the vascular tone and have an impact on NOS/HO systems; thus, their effects were analysed in the cardiac tissue of OVX and SO rats as well as in that of animals treated with estrogen (OVX + E_2_) or with both AEA and estrogen (OVX + E_2_ + AEA).

## 2. Results

### 2.1. NOS Activity and eNOS Expression

**Figure 1:**
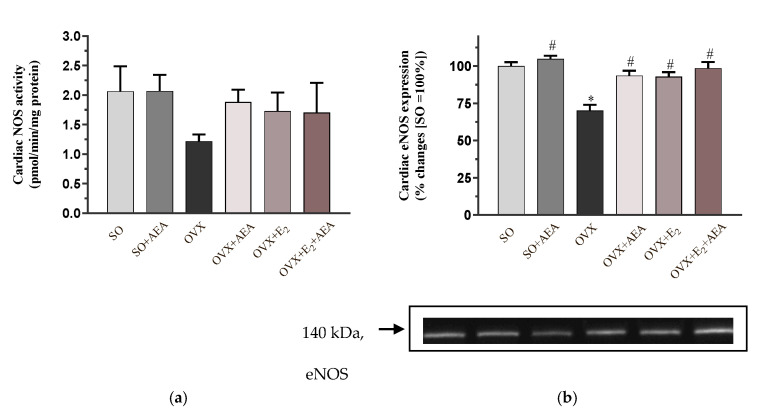
Effects of anandamide and estrogen treatments on cardiac activity and expression of the NOS enzyme in SO and OVX animals. (**a**) Cardiac NOS activity (expressed as pmol/min/mg protein); results are shown as means ± S.E.M. *n* = 6–8; (**b**) Representative image of cardiac eNOS expression (expressed as % changes, where SO = 100%); results are shown as means ± S.E.M. *n* = 5–9; * *p* < 0.05: statistical significance relative to the SO group, # *p* < 0.05: statistical significance relative to the OVX group. SO = sham operation, OVX = surgical ovariectomy, AEA = anandamide, E_2_ = estrogen, NOS = nitric oxide synthase, eNOS = endothelial nitric oxide synthase.

As shown in Figure 1a, AEA treatment did not cause any changes in cardiac NOS activity in SO animals. NOS activity values were reduced in estrogen-depleted animals; however, two weeks of AEA treatment improved the OVX-induced unfavorable alterations. A similar compensatory effect was observed in OVX rats treated with E_2_ and with E_2_ + AEA.

When we analyzed the expression of the eNOS isoform (i.e., the most potent cardiovascular regulator of NOS isoforms), we observed that its expression level in OVX rats was only 70% of the expression level in the SO group. The pharmacological manipulation of eNOS level by AEA or E_2_ administration resulted in a significant improvement in eNOS values. Similar to these effects of the individual compounds, co-treatment of AEA and E_2_ also had a beneficial impact on cardiac regulation, although a further increase in eNOS expression was not observed. The results are shown in Figure 1b.

### 2.2. HO Activity and HO-1 Expression

**Figure 2:**
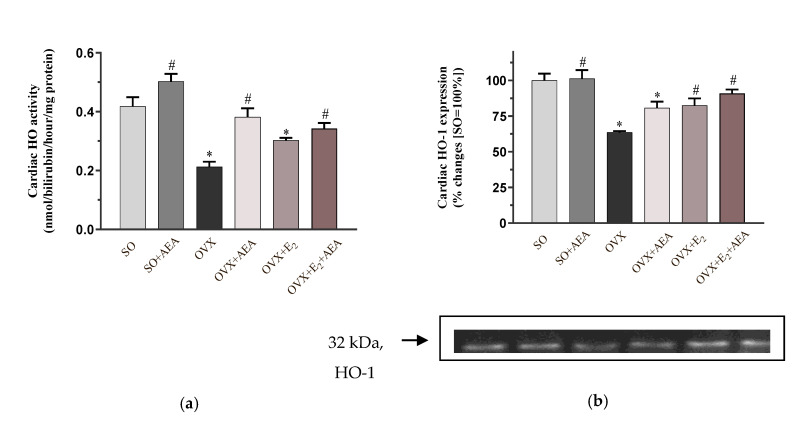
Effects of anandamide and estrogen treatment on cardiac activity and expression of the HO enzyme in SO and OVX animals. (**a**) Cardiac HO activity (expressed as pmol/min/mg protein); results are shown as means ± S.E.M. *n* = 7–9; (**b**) representative image of cardiac HO-1 expression (expressed as % changes, where SO = 100%); results are shown as means ± S.E.M. *n* = 5–8; * *p* < 0.05: statistical significance relative to the SO group, # *p* < 0.05: statistical significance relative to the OVX group. HO = heme oxygenase, HO-1 = heme oxygenase-1.

To analyze the potential involvement of the HO system in the AEA-induced cardiovascular effects, the activity and expression of the HO-1 isoform were measured. Our results clearly show that OVX-induced estrogen loss resulted in a significant decrease in the activity of HO, which was significantly restored by either AEA or E_2_ treatment. However, the combined use of AEA and E_2_ did not cause a further enhancement in the enzyme activity. The results are shown in Figure 2a.

Similar to the changes of HO activity, we did not find differences between SO and SO + AEA groups. Our results clearly show that the HO-1 isoform was significantly reduced in OVX animals compared to the SO (fertile control) group. Two weeks of AEA or E_2_ treatment significantly enhanced the level of HO-1 expression in both OVX + E_2_ and OVX + E_2_ + AEA groups compared to the OVX animals. The results are shown in Figure 2b.

### 2.3. Cardiac CB1 Receptor Content

**Figure 3:**
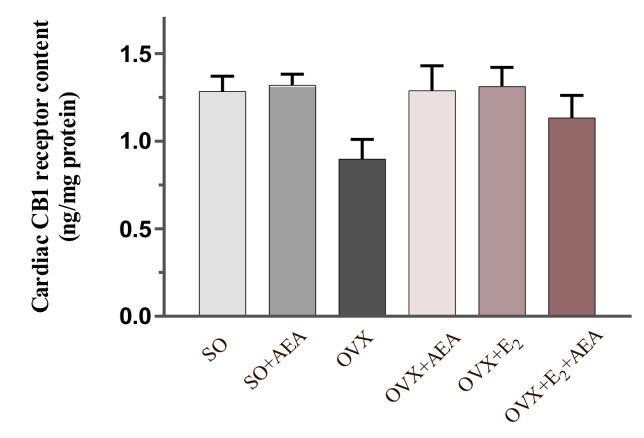
Effects of anandamide and estrogen treatment on cardiac CB1 receptor content in SO and OVX animals (expressed as ng/mg protein). Results are shown as means ± S.E.M; *n* = 5–11; CB1 receptor = cannabinoid 1 receptor.

Figure 3 shows that the content of CB1 receptor was slightly diminished in the cardiac tissue of estrogen-deficient females. However, two weeks of AEA and E_2_ treatment compensated the decreased content of CB1 receptor.

### 2.4. Cardiac cGMP Concentration

**Figure 4:**
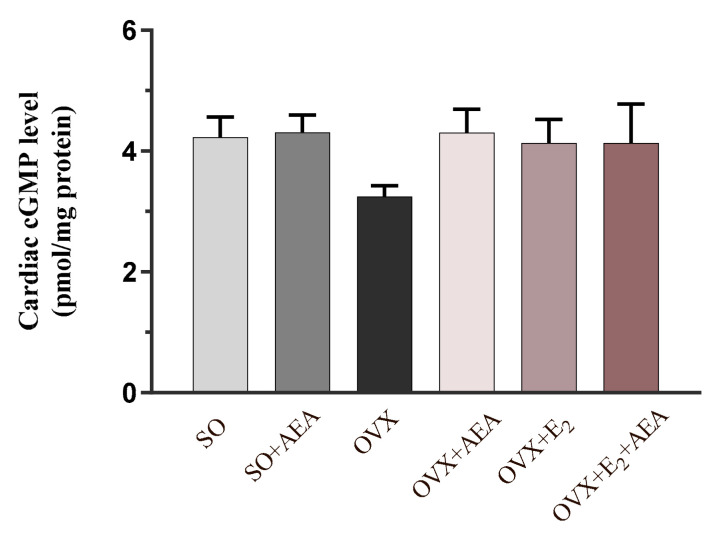
Effects of anandamide and estrogen treatment on cardiac cGMP levels in SO and OVX animals (expressed as pmol/mg protein). Results are shown as means ± S.E.M. *n* = 5–10; cGMP = cyclic guanosine monophosphate.

To verify the potential vasorelaxant role of NOS and HO systems, the concentration of the second messenger cGMP was also measured. As expected, OVX rats exhibited the lowest concentration of cGMP, which was augmented by AEA and E_2_ treatment. Figure 4 shows that AEA treatment resulted in similar cGMP levels in both SO and OVX groups, which were equivalent to the cGMP values of E_2_-treated and (E_2_ + AEA)-treated OVX animals.

### 2.5. Cardiac TRPV1 and CGRP Concentrations

**Figure 5:**
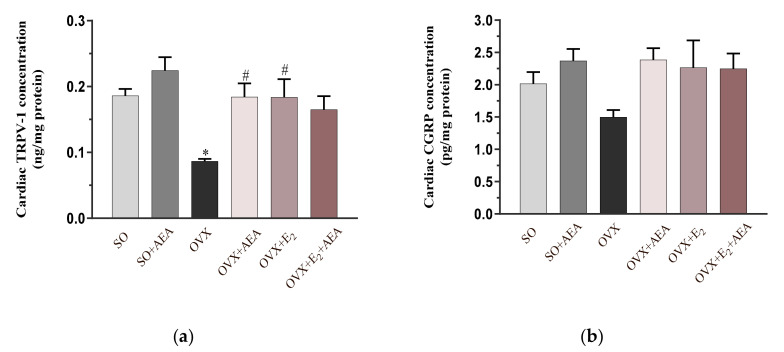
Effects of anandamide and estrogen treatment on cardiac TRPV1 and CGRP concentrations in SO and OVX animals. (**a**) Cardiac TRPV1 concentration (expressed as ng/mg protein); results are shown as means ± S.E.M. *n* = 5–9; (**b**) cardiac CGRP concentration (expressed pg/mg protein; results are shown as means ± S.E.M. *n* = 6–11; * *p* < 0.05: statistical significance relative to the SO group, # *p* < 0.05: statistical significance relative to the OVX group. TRPV1 = transient receptor potential vanilloid 1, CGRP = calcitonin gene related peptide.

Our results prove that TRPV1/CGRP pathways play a role in AEA-induced cardiovascular changes. As shown in Figure 5a, TRPV1 concentration was significantly decreased in OVX rats. In these animals, the individual use of AEA and E_2_ caused a ~50% increase in TRPV1 concentration; however, co-treatment with AEA and E_2_ did not produce a further enhancement.

In accordance with the positive cardiovascular effects of CGRP, we found that AEA and E_2_ treatments improved the cardiac concentration of CGRP, which was diminished as a result of estrogen withdrawal. Figure 5b shows that CGRP concentration was similar in SO and OVX animals and in groups treated with AEA-, E_2_, or E_2_ + AEA.

## 3. Discussion

Since estrogen deficiency is strongly associated with an increased risk of CVDs and a shorter lifespan in postmenopausal women, understanding the underlying mechanisms as well as finding therapeutic approaches for cardioprotection have received significant attention and need to be investigated more deeply. Taking into account the ‘timing hypothesis’, there are abundant data supporting estrogen hormone therapy as a protective approach against CVDs [5,13]. As myocardial protection includes complex mechanisms, therapeutically targeting multiple factors (receptors, signaling cascades, and enzymatic activators/inhibitors) could contribute to improving cardiovascular mechanisms [14,15,16]. Our results support the fact that the endocannabinoid system plays a role in the regulation of cardiovascular signaling pathways and could be a therapeutic target for menopause-associated cardiac pathologies.

The main finding of this study is that administration of the endocannabinoid AEA restored the estrogen loss-induced adverse effects via the NOS/HO systems and the activation of the TRPV1/CGRP pathway. The similarity between AEA- and estrogen-induced changes suggests estrogen-like effects of AEA in estrogen-depleted conditions. However, the combined use of AEA and E_2_ was not able to amplify the individual estrogen or AEA actions.

Endogenous estrogen loss leads to a series of biochemical, morphological, and functional changes, among which increased oxidative and inflammatory processes, accumulation of collagen deposition, as well as increased blood pressure and aortic contraction [17,18]. In our previous studies, we proved that two weeks of low-dose HRT restored the pathological changes in both cardiac and aortic tissues via the HO system [5,19]. The HO family consists of three isoforms, including inducible HO-1 and the constitutive HO-2 and HO-3 isoforms [20]. There is evidence that HO mediates adaptive responses and contributes to the maintenance of cardiovascular health. The HO/CO signaling pathway plays a physiological role in the myocardium and vasculature, which suggests its potential significance in the pathogenesis of CVDs, including menopause-associated atherosclerosis, hypertension, and cardiac remodeling [16,21,22]. In accordance with our earlier observations, we found that OVX-induced estrogen withdrawal resulted in a significant decrease in the activity and expression of HO-1 compared to the SO group. Similar to the decrease of HO, NOS enzyme activity and expression were also diminished in OVX animals. Decreased activity of NOS and loss of NO bioavailability are potential contributors to cardiovascular complications. Physiologically, NO is formed by NOS by converting L-arginine to citrulline. Three isoforms of the family (endothelial NOS/eNOS, neuronal NOS/nNOS, and inducible NOS/iNOS) have been identified. Within the cardiovascular system, eNOS plays a crucial role in antioxidant mechanisms and regulates a wide spectrum of vascular functions, including vasorelaxation, inhibition of leukocyte adhesion, and migration and proliferation of vascular smooth muscle cells [23]. Growing evidence indicates that reactive oxygen species (ROS)-related processes and conditions, such as menopause, alter antioxidant/oxidant homeostasis in the cardiovascular system, thus making the heart more vulnerable to the development of CVDs, which contribute to endothelial dysfunction [24]. In this scenario, decreased activity of the HO/NOS systems and therefore of their vasorelaxant effects can be associated with OVX-induced ROS overproduction in the heart. To compensate the adverse cardiac effects of estrogen deficiency, plausible cardioprotective signaling cascades were examined. Beside estrogen replacement therapy, which is well-described in the literature, endocannabinoids possess pleiotropic effects on the cardiovascular system [25]. While activation of the endocannabinoid system plays a minimal role in the regulation of cardiac function during physiological circumstances, its increased activation could be detrimental or beneficial in various pathological conditions, as extensively investigated and proved with high-quality results by the research group of Pacher and Batkai [26,27]. Pacher et al. reported that endocannabinoids play an important role in hemorrhagic, endotoxic, and cardiogenic shock as well as in liver cirrhosis [28]. In addition, studies in animal models proved that stimulation of the CB1 receptor mediates pathological mechanisms, including several cell death pathways, oxidative stress, vascular inflammatory responses, and therefore cardiac dysfunction [29,30]. Beside these unfavurable effects, targeting the ECS is beneficial for regulating and maintaining the blood pressure in different models of hypertension. Due to the broad range of complications and pathological alterations in postmenopausal women, estrogen replacement therapy is a traditional pharmaceutical approach to effectively address specific cardiovascular risk factors. In the context of HRT, timing (i.e., start of the HRT), duration, and hormonal dose show strong correlation with CVD risk and further chronic disease, such as neoplastic changes and neurological disorders [31]. Although both HRT and AEA treatments possess advantageous and disadvantageous effects, the examination of AEA-induced pathways may provide new targets and mechanisms of hemodynamic regulation. In this regard, Underdown et al. found that AEA treatment reduced infarct size in rat isolated heart by interaction with one or more mechanisms of cannabinoid action [32]. Tuma and Steffens summarized that the mechanisms by which endocannabinoids are cardioprotective include decreased inflammation and oxidative stress as well as increased activation of cardioprotective signaling pathways through activation of CB1 and CB2 receptors [33]. CB1 receptors are predominantly expressed in the central nervous system but also present at much lower levels in the myocardium, postganglionic autonomic nerve terminals, and vascular endothelial and smooth muscle cells [27]. Our current findings show that the presence of CB1 receptors was reduced in the cardiac tissue of the OVX animals; however, AEA treatment restored it. In addition, both the activity and the expression of NOS and HO systems also diminished in the presence of CB1R; however, these unfavorable changes were compensated by two weeks of AEA treatment. In the literature, previous studies verified the role of AEA in the regulation of vascular tone. Romano and Lograno as well as Stanley, Hind, and O’Sullivan documented that AEA induces endothelium-dependent relaxation via CB1R-mediated NOS activation [11,34]. In the cardiovascular system, NO exerts its antioxidant, antiplatelet, and vasorelaxant properties via the NO/cGMP pathway. NO activates sGC to produce the second messenger cGMP, which results in vasodilatation. Consequently, a decline in NO bioavailability increases the risk of CVDs, which is mainly due to the inactivation of NO by excessive production of superoxide anion radicals. Many studies support the strong correlation between HO/CO and NOS/NO systems and prove that the HO enzyme and heme degradation products improve the vascular function, at least in part, by preserving NO bioavailability. Pae et al. summarized the three possible scenarios for preserving NO level via HO-1. These are modulating the expression and activity of eNOS, preventing the inactivation of vascular NO, and compensating the loss of NO [9]. Our recent findings clearly show that both AEA and E_2_ treatments compensated OVX-induced reduction in both NOS/eNOS and HO/HO-1 levels. HO acts in many ways that are similar to the action of vascular NO, including the sGC/cGMP-mediated vasorelaxant effects. Our results support this hypothesis, as estrogen withdrawal resulted in a decrease in cardiac cGMP concentration; however, AEA and E_2_ treatments compensated this reduction, similar to the observed NOS/NO and HO/CO changes.

Accumulating evidence indicates that AEA-induced vasorelaxation and cardioprotection can be mediated via alternative receptors, such as TRPV1. TRPV1 is located predominantly in nerve fibers that innervate the cardiovascular system, and its activation stimulates the release of a dilator neuropeptide, CGRP. In a previous study, Ross analyzed the interaction of AEA with TRPV1 and reported that AEA-induced CGRP release may induce a potential endogenous myocardial protective response [35]. Our results clearly showed that the TRPV1/CGRP system was underactive in estrogen-depleted conditions; however, these unfavorable changes were reversed as a result of both AEA and estrogen treatment. Furthermore, our data proved that AEA and estrogen treatments not only upregulate the activity and expressions of HO and NOS enzymes, but also induce CGRP release. In accordance with Peng et al. who examined the co-mediated effects of HO and CGRP, we can presume a potential interaction in biological effects of CO, NO, and CGRP [36].

**Figure 6:**
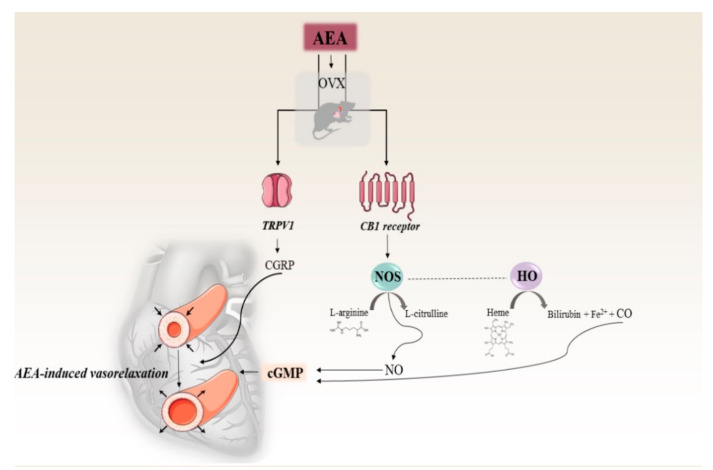
Effects of anandamide on NOS/HO systems, and TRPV1/CGRP pathway in an estrogen-deficient rat model. short arrows indicate changes of the diameter of the vessels. AEA= anadamide, OVX= surgical ovariectomy, TRPV1= transient receptor potential vanilloid 1, CGRP= calcitonin gene related peptide, CB1 receptor= cannabinoid 1 receptor, cGMP= cyclic guanosine monophosphate, NOS= nitric oxide synthase, HO= heme oxygenase, NO= nitric oxide, CO= carbon monoxide.

An overview of the AEA-induced cardiac effects on NOS/HO systems and the TRPV1/CGRP pathway in estrogen-deficient conditions is summarized in Figure 6.

### Limitations

In our study, we focused on AEA-induced cardiac effects on HO/NOS systems; however, metabolic changes and functional parameters were not studied. In order to get a deeper insight into AEA-induced complex cardiovascular mechanisms related to estrogen-deficient pathologies, further investigations are needed.

## 4. Materials and Methods

### 4.1. Animals

In our study, 180–200 g female Wistar rats (Toxi-Coop Zrt., Budapest, Hungary) were used and housed (Directive 2010/63/EU) at controlled temperature (20–23 °C) on a 12 h light–dark cycle. Phytoestrogen-free chow and tap water were available ad libitum. All procedures were approved by the Institutional Ethics Committee (approval code: XX/2018.) and were performed in accordance with the standards of the European Community guidelines on the care and use of laboratory animals (XX./2633/2020.).

### 4.2. Experimental Design

Following a one-week acclimatization period, rats were subjected to either bilateral OVX or SO under anesthesia with thiopental (5 mg/100 g, intraperitoneally (i.p.)). During OVX, a bilateral dorsolateral incision was made in the skin and muscular layer for the removal of the ovaries and ligation of the uterine tubes. On the contrary, the SO group underwent the same surgical procedure with exposure of the ovaries, which were then returned to their original position. After OVX and SO, the wound was treated with penicillin, then the muscle layers and skin were sutured.

After a 4-week resting period, one part of OVX animals were pretreated per os with 0.1 mg/kg estrogen (E_2_, Estrofem, Novo Nordisk A/S, Bagsvaerd, Denmark) once daily, for two weeks (OVX + E_2_). The protocol for estrogen hormone replacement was based on our previous study, which demonstrated that this dose is capable to maintain the physiological concentration of E_2_ observed in controls [5].

In order to detect AEA-induced cardiovascular effects, SO, OVX, and some OVX + E_2_ rats received intraperitoneal AEA injection at a daily dose of 1.0 mg/kg, for a total of two weeks; in this way SO + AEA and OVX + AEA subgroups were defined. In the group of rats who received both estrogen pretreatment and AEA injection (OVX + E_2_ + AEA), estrogen replacement was continuous during the AEA treatment period. Animals who did not receive AEA injection were treated with the same volume of vehicle (0.2 mL). At the end of the experiment, cardiac samples were excised, frozen, and kept at −80 °C until biochemical analysis. In our study, all efforts were made to minimize the number of animals as well as their suffering.

**Figure 7:**
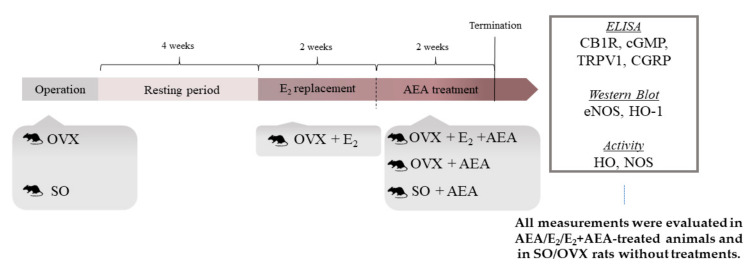
Experimental design of the study.

The experimental design of the study is presented in Figure 7.

### 4.3. Measurement of NOS Activity

NOS activity was determined via a NOS activity Assay Kit (Abcam, ab211083). Frozen heart samples were homogenized in cold NOS Assay Buffer and centrifuged for 10 min at 4 °C at 10,000× *g*. Supernatants were collected, from which protein amounts were measured via the Bradford method. Protein determination was immediately followed by NOS activity assay. First, a NOS Reaction Mix was prepared from the following components: diluted NOS Cofactor I, NOS Cofactor 2, NOS Substrate, and Nitrate Reductase. Preparation of the standard was performed as described in the protocol; 60 µL of each substance was pipetted into the wells; the sample wells were filled with 40 µL of the previously mentioned supernatants; 40 µL of the Reaction Mix was added into standard and sample wells, which was followed by a 1 h incubation at 37 °C. After incubation, 95 µL of NOS Assay Buffer and 5 µL of Enhancer were added to standard and sample wells. This reaction mixture was incubated for another 10 min at room temperature. For the color reaction, 50 µL of Griess Reagent I and II were pipetted into standard and sample wells. Absorbance was measured after a 10 min incubation time via a microplate reader at OD 540 nm. NOS specific activity was then calculated as NOS specific activity = (B/T*C), where: B = amount of nitrite amount in the sample well calculated from a Standard Curve (pmol), T = reaction time (minutes) − 60 min in this case, C = amount of protein (µg).

### 4.4. Measurement of HO Activity

Cardiac tissues were homogenized in a cold buffer (pH 7.4, 10.0 mM N-2-hydroxyethylpiperazine-N’-2-ethanesulfonic acid, 10.0 μg/mL leupeptin; 0.10 mM ethylenediaminetetraacetic acid disodium salt dihydrate, 32.0 mM sucrose, 10.0 μg/mL trypsin inhibitor, 1.0 mM dithiotreitol (DTT), 2.0 μg/mL aprotinin), centrifuged at 15,000× *g* for 20 min at 4 °C. The supernatant was used for the determination of HO activity by two parallel measurements, namely, a blind measurement and an NADPH measurement. The reaction mix contained 75 µL of sample, 2.0 mM glucose-6-phosphate, 0.14 U/mL glucose-6-phosphate dehydrogenase, 15.0 μM hemin, 120.0 μg/mL rat biliverdin reductase, and a complex buffer composed of 100.0 mM KH2PO4, phenylmethylsulfonyl fluoride, 2.0 μg/mL aprotinin, 10.0 μg/mL trypsin inhibitor, 10.0 μg/mL leupeptin, 2.0 mM MgCl_2_ × 6H_2_O, and 1.0 mM DTT. During NADPH measurements, 100 µL of reduced β-nicotinamide adenine dinucletotide phosphate (β-NADPH) was added into the mixture in order to initiate the reaction, followed by incubation at 37 °C for 60 min. After incubation, ice cooling was applied to stop the reaction. For the blind measurement, β-NADPH was replaced with the measuring buffer. Using a microplate reader, the NADPH and blind solutions were both measured at 465 nm spectrophotometrically. In order to obtain HO activity, the blind values had to be subtracted from the NADPH values. HO activity was represented as the hourly produced amount of bilirubin in nmol/mg protein.

### 4.5. Measurement of eNOS and HO-1 Expression

Cardiac tissues were homogenized in ice-cold homo buffer (containing phosphatase inhibitor, vanadate (1:50)) and radio immunoprecipitation assay (RIPA) buffer (containing a protease inhibitor and TRITON-X-100) for eNOS and HO-1 proteins, respectively. Homogenates were centrifuged at 12,000 r.p.m. at 4 °C for 10 min, boiled for 5 min, and then the protein concentration was determined by the Bradford method. Equal amounts of protein (50 µg) were analyzed through electrophoresis (90 V/2 gels) on 8% and 10% polyacrylamide gel for eNOS and HO-1, respectively. Then, the proteins were transferred to polyvinylidene fluoride (PVDF) membranes overnight at 25 V for eNOS and for 2.5 h at 30 V for HO-1. In the presence of 0.10% Ponceau red, we could determine the equivalence of protein loading. To increase the final signal sensitivity, the membranes were incubated with Pierce Western Blot Signal Enhancer (Thermo Fisher Scientific, Waltham, MA, USA) and then blocked in 5% non-fat dry milk in TBS–Tween buffer. The blocking period was 2 h at room temperature followed by 3.5 h at 4 °C for eNOS and overnight incubation at 4 °C for HO-1. The blots were probed overnight (4 °C, 1% milk) with anti-eNOS mouse primary antibody (1:500, ab76198, Abcam, Cambridge, UK) or anti-HO-1 mouse primary antibody (1:500, ADI-OSA-111, Enzo Life Sciences). The membranes were then incubated for 1 h at room temperature with a secondary anti-mouse antibody (1:4000, Dako, Glostrup, Denmark A/S) for both eNOS and HO-1. MagicMark XP Western protein standard (Invitrogen, Thermo Fisher Scientific, Waltham, MA, USA) was used for convenient protein determination. The standard consists of 9 recombinant proteins ranging in molecular weight from 20 to 220 kDa and allowed the identification of both eNOS and HO-1. The bands were visualized by Uvi Chemi Pro. and analyzed by Quantity One software (Bio-Rad, Hercules, CA, USA).

### 4.6. Determination of Cardiac CB1 Receptor, cGMP, TRPV1, and CGRP Concentrations

Heart tissues were homogenized in phosphate buffer (PBS) (pH 7.4) for 30 s and centrifuged for 20 min at 3000 rpm, 4 °C. Supernatants were collected, from which protein amounts were measured via the Bradford method. Protein determination was followed by enzyme-linked immune sorbent assay (ELISA; GenAsia, Shanghai). Standard solutions were prepared as described in the manufacturer’s protocol and pipetted into the wells together with 50 of µL streptavidin–HRP. Sample wells were filled with 40 µL of supernatants, 10 µL of antibodies, and 50 µL of streptavidin–HRP. Using a laboratory plate incubator, the plate was gently shaken and incubated for 60 min at 37 °C. Afterwards, the plate was carefully washed 5 times, and chromogen solutions A and B were added to the wells, which was followed by a 10 min incubation at 37 °C. At the end, a stop solution was added to each well, and absorbance (OD) was measured at 450 nm wavelength. CB1 receptor, cGMP, TRPV1, and CGRP levels were expressed as ng/mg, pmol/mg, ng/mg, and pg/mg protein, respectively.

### 4.7. Statistical Analysis

Data are presented as the average outcome in a group (mean) +/− standard error of the mean (SEM). The Shapiro–Wilk normality test was used to estimate a Gaussian distribution, and statistical analysis was then performed using one-way analysis of variance (ANOVA) followed by Tukey post-test (if the normality test was passed) or Kruskal–Wallis test followed by Dunn’s post-test (if the normality test was not passed). Statistics were performed using GraphPad Prism for Windows, version 7.00 (GraphPad Software Inc., La Jolla, CA, USA). Probability values (*p*) less than 0.05 were considered significantly different.

## 5. Conclusions

A large body of evidence now exists indicating that the endocannabinoid system plays an important role in the cardiovascular system; however, its regulatory role is controversial and not fully elucidated. Depending on the underlying pathology, the activation of CB1 and TRPV1 receptors may offer therapeutic benefits. In our study, we analyzed the cardiovascular effects of NOS/NO, HO/CO, and TRPV1/CGRP in different estrogen-saturated conditions. In this scenario, we proved that two weeks of either AEA or E_2_ treatment enhanced the NO and HO enzyme systems via CB1R activation, and this tendency was also shown for the TRPV1/CGRP pathway. Furthermore, AEA-induced alterations were similar to the effects of the estrogen replacement therapy, which suggests AEA estrogen-like role in this model.

Our findings may help decipher the complexity of the endocannabinoid system in cardiovascular regulation related to estrogen-depleted conditions, which may lead to the identification of new target sites of action for drug treatment.

## Abbreviations

AEA	anandamide
CB1R	cannabinoid 1 receptor
cGMP	cyclic guanosine monophosphate
CGRP	calcitonin gene related peptide
CO	carbon monoxide
E_2_	estrogen replacement therapy
eNOS	endothelial nitric oxide synthase
HO	heme oxygenase
NO	nitric oxide
NOS	nitric oxide synthase
sGC	soluble guanylate cyclase
SO	sham operation
OVX	surgical ovariectomy
TRPV1	transient potential vanilloid receptor 1
